# Association between serum uric acid and carotid atherosclerosis in elderly postmenopausal women: A hospital‐based study

**DOI:** 10.1002/jcla.24097

**Published:** 2021-11-27

**Authors:** Xiao‐kang Dong, Dan Luo, Wen‐jing Chen, Rong‐rong Wang, Jie Yang, Miao‐miao Niu

**Affiliations:** ^1^ Department of Cardiovascular Affiliated Hospital of Shandong University of Traditional Chinese Medicine Jinan China; ^2^ Department of Endocrinology Affiliated Hospital of Shandong University of Traditional Chinese Medicine Jinan China; ^3^ Department of Gynecology Sishui County People's Hospital Jining China; ^4^ Department of Pharmacy Tai'an Traditional Second Chinese Medicine Hospital Tai'an China

**Keywords:** carotid atherosclerosis, hormone replacement therapy, serum uric acid

## Abstract

**Background:**

Carotid atherosclerosis (CAS) is associated with increased cardiovascular risk and implicated in 20–30% of strokes.

**Methods:**

504 patients were included in this study. The detailed medical history and the results of physical examination, carotid ultrasound examination, and routine laboratory tests were collected. Logistic regression analyses were conducted to analyze the relationship between the SUA and the presence of carotid plaques. And the relationship between SUA and the progression of CAS was analyzed by multiple linear regression. The effect of hormone replacement therapy (HRT) on CAS has also be evaluated.

**Results:**

412 patients (81.7%) had carotid plaques of different sizes by carotid ultrasound examination. We found a positive association between the level of SUA and the probability of having carotid plaque by univariate logistic regression (OR: 2.01, 95% CI: 1.83–2.19, *p* = 0.003). At 2 years post‐discharge, we found that 1 mg/dL increase in SUA levels was expected to 0.946% increase in plaque score and 0.026 cm increase in carotid intima‐media thickness, separately. Moreover, patients treated by long‐term HRT (≥5 years) had a lower level of SUA and blood lipid and the less change of plaque score and carotid intima‐media thickness than patients without HRT.

**Conclusion:**

The presence and progression of CAS had significantly positive associations with the level of SUA. And the HRT may have the ability to prevent the presence and progression of CAS. However, the safety and long‐term outcome of HRT on CAS should be evaluated in further studies.

## Graphical abstract

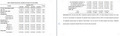

## 1 INTRODUCTION

Atherosclerosis, a chronic immunoinflammatory disease of blood vessels, is frequently occurred in middle‐aged and aged people. It results in deaths of approximately 20 million per year, according to the World Health Organization (WHO).^1^ As a kind of atherosclerosis, subclinical atherosclerosis (SA) refers to the pathological changes of bilateral common carotid artery, common carotid artery bifurcation, and extracranial segment of internal carotid artery, such as tube wall stiffness, intima‐media thickening, subintimal lipid deposition, plaque or ulcer formation, and lumen stenosis.^2^ Carotid intima‐media thickness (CIMT) and carotid atherosclerosis (CAS) plaques are common and reliable methods for evaluating the SA.

Many risk factors were reported to be associated with atherosclerosis, such as retinal vascular abnormalities,^3^ obesity,^4^ diabetes,^5^ hypercholesterolemia,^6^ hyperhomocysteinemia,^7^ and hyperlipidemia.^8^ In recent years, serum uric acid (SUA), as the natural product of purine metabolism, has been confirmed to be associated with the risk of atherosclerosis.^9^ Increased SUA was associated with the development and progression of atherosclerosis.^10^ However, the researches focused on the relationship between SUA and SA was still absent. Furthermore, studies have shown that menopausal women are more likely to develop atherosclerosis, especially prominent in late postmenopausal women. Intriguingly, the serum uric acid levels had obviously an age‐associated increase among women.^11^, ^12^, ^13^ Nevertheless, no reports documented the relationship between the levels of serum uric acid and atherosclerosis, especially SA. Hormone replacement therapy (HRT) refers to injecting drugs containing missing hormones into patients through intravenous injection to replace missing hormones.^14^

In this study, we mainly investigate the relationship between the level of serum uric acid and the presence and progression of SA in Chinese postmenopausal women. And the benefits of HRT on atherosclerosis in postmenopausal women have also be evaluated. Hopefully, our findings could be used in the early prognosis and timely intervention of SA for postmenopausal women.

## 2 METHODS

### 2.1 Study participants

This study enrolled female patients over 70 years old in our outpatient clinical center. These patients visited our outpatient department and were diagnosed by CAS from May 2016 to December 2019. All participants provided detailed medical history and received physical examinations, carotid ultrasound examination, and routine laboratory tests. Follow‐up of the patients after outpatient visits was performed at 6^th^, 12^th^, and 24^th^ months. The carotid ultrasound examination and serological examination, including SUA tests, were repeated in outpatient reviews at 24^th^ month. Patients who underwent drug treatment for uric acid control or had stroke histories were excluded from this study.

And they all signed a written informed consent form. Ethical approval was obtained from the Ethics Committee of the Affiliated Hospital of Shandong University of Traditional Chinese Medicine.

### 2.2 Laboratory measurements

General serological tests were performed for all participants. Routine blood biochemistry profile included blood glucose, liver and kidney function, serum uric acid, and blood lipids, including high‐density lipoprotein cholesterol (HDL‐C), low‐density lipoprotein cholesterol (LDL‐C), total cholesterol (TC), and triglyceride (TG) (Guilin URIT Medical Electronics Co., Ltd.). The blood glucose was measured by a Roche blood glucose meter (ROCHE). And the liver and renal function, serum uric acid, and lipid profile were tested using an automatic biochemical analyzer (ERBA Diagnostics Mannheim GmbH). The measurements of these parameters on the first outpatient visits and during the outpatient reviews were used in our analysis.

### 2.3 Carotid ultrasound

Carotid ultrasound examination was also performed in all patients via the ultrasound system (GE LOGIQ 400 PRO; GE) of our hospital. A trained and certified sonographer conducted such examination for all patients in the supine position. It was used to quantify CIMT for the patients in order to evaluate the severity of extracranial carotid atherosclerosis. The presence of carotid plaque and thickness of carotid plaques was assessed for each individual. The plaque was defined as clearly isolated focal thickening (≥1.1 mm in thickness).^15^ The plaque score was calculated by summing the plaque thicknesses of all segments.

Patients received the carotid ultrasound examination again in outpatient reviews at 24^th^ month. The ratio of absolute difference of plaque score between the first examination upon hospitalization and the examination 24 months after discharge was calculated. And the absolute difference of carotid intima‐media thickness between the two examinations was calculated as well.

### 2.4 Statistical analysis

The normally distributed data were presented as means ± standard errors. The frequency and percentage (%) were used for categorical variables. The continuous variables were analyzed by analysis of variance (ANOVA). For the ordered categorical variables, the Mann–Whitney *U* test was used to compare two independent groups, and the Kruskal–Wallis test was used in the comparisons of three independent groups.

The association between the levels of SUA and the presence of carotid plaque was analyzed by binary logistic regression. And the association between the level of SUA and the progression of CAS was analyzed by univariate and multivariate logistic regression analysis. In this model, the percentage of change of plaque score and the absolute difference of carotid intima‐media thickness between the first examination upon hospitalization and the examination 24 months after discharge were set as the dependent variable, separately. Moreover, in the patients with plaques, the effect of HRT was also investigated by comparing the levels of SUA, the percentage of change of plaque score, and the change of carotid intima‐media thickness. All statistical analyses were performed by SPSS software (SPSS version 22.0, SPSS, Inc.).

## 3 RESULTS

### 3.1 Baseline characteristics of patients

In total, 504 patients were included in this study. Table 1 shows the baseline characteristics of these patients by SUA quartiles. The elderly female patients who had higher SUA levels were more likely to have older age, higher BMI, hypertension, diabetes mellitus, and relatively higher levels of TC, TG, LDL‐C, and the relatively lower levels of HDL‐C. Moreover, increased SUA was associated with less frequent use of HRT (*p* < 0.05), but it was related to the higher incidence of carotid plaque (*p* < 0.01). And there were 412 patients (81.7%) had carotid plaques of different sizes by carotid ultrasound examination.

**TABLE 1:** Baseline characteristics of patients according to uric acid quartiles

Variables	Quartiles of SUA				*p* value
	1 (*n* = 125)	2 (*n* = 126)	3 (*n* = 126)	4 (*n* = 127)	
Uric acid range (median), mg/dL	4.8–5.6 (5.2)	5.7–7.5 (6.1)	7.6–8.3 (8.0)	8.4–9.2 (8.7)	
Age, years	71.25 ± 5.32	72.75 ± 4.79	73.18 ± 4.26	74.56 ± 5.03	<0.001
Body mass index, kg/m^2^	24.15 ± 3.12	24.23 ± 2.62	24.39 ± 2.71	24.63 ± 2.45	0.012
Drinking, %	25 (20.0%)	28 (22.2%)	31 (24.6%)	33 (26.0%)	0.324
Smoking status					
Nonsmokers (%)	65 (52.0%)	62 (49.2%)	68 (54.0%)	56 (44.1%)	0.051
Former smokers (%)	28 (22.4%)	29 (23.0%)	21 (16.7%)	32 (25.2%)	0.032
Current smokers (%)	32 (25.6%)	35 (27.8%)	37 (29.4%)	39 (30.7%)	0.008
Hypertension, %	40 (32.0%)	45 (35.7%)	51 (40.5%)	53 (41.7%)	0.014
Diabetes mellitus, %	25 (20.0%)	29 (23.0%)	31 (24.6%)	27 (21.3%)	0.041
Serum lipid data					
High TC, %	25 (20.0%)	23 (18.3%)	19 (15.1%)	28 (22.0%)	0.025
High TG, %	31 (24.8%)	35 (27.8%)	38 (30.2%)	39 (30.7%)	0.034
High LDL‐C, %	35 (28.0%)	40 (31.7%)	41 (32.5%)	43 (33.9%)	0.035
Low HDL‐C, %	34 (27.2%)	39 (31.0%)	43 (34.1%)	44 (34.6%)	0.021
Drugs history					
Use of statin, %	23 (18.4%)	25 (19.8%)	26 (20.6%)	24 (18.9%)	0.175
Use of antihypertensive medication, %	31 (24.8%)	34 (27.0%)	40 (31.7%)	46 (36.2%)	0.163
Use of antidiabetic medication, %	18 (14.4%)	24 (19.0%)	25 (19.8%)	21 (16.5%)	0.271
Use of hormone replacement therapy, %	62 (49.6%)	55 (43.7%)	52 (41.3%)	45 (35.4%)	0.038
Cardiovascular disease history, %	13 (10.4%)	15 (11.9%)	17 (13.5%)	20 (11.0%)	0.038
Carotid plaque, %	95 (76.0%)	102 (81.0%)	103 (81.7%)	112 (88.2%)	0.003
CIMT, mm					
<1 mm	112 (89.6%)	120 (95.2%)	122 (96.8%)	126 (99.2%)	0.025
≥1 mm	13 (10.4%)	6 (4.8%)	4 (3.2%)	1 (0.8%)	

Note

The quantitative data was analyzed by One‐way ANOVA. The ordered categorical data between two independent exposure groups was analyzed by Mann–Whitney *U* test was used for. The ordered categorical data among 3 independent exposure groups was analyzed by Kruskal‐Wallis test. *p* values for trend was presented.

Abbreviations: BMI, body mass index; HDL‐C, high‐density lipoprotein cholesterol; LDL‐C, low density lipoprotein cholesterol; SUA, serum uric acid; TC, total cholesterol; TG, triglyceride.

### 3.2 The level of SUA and the incidence of carotid plaque

Among the postmenopausal women aged over 70 years, we found the positive association between the level of SUA and the probability of having carotid plaque by univariate logistic regression (OR: 2.01, 95% CI: 1.83–2.19, *p* = 0.003). After adjusting the age, such association remained significant but mildly decreased (OR: 1.95, 95% CI: 1.81–2.09, *p* = 0.013). Further adjustment for smoking, drinking, hypertension, diabetes mellitus, and the drug history of statin, antihypertensive medication, antidiabetic medication, and HRT attenuated the association (OR: 1.83, 95% CI: 1.56–2.11, *p* = 0.025). And additional adjustments for TC, TG, LDL‐C, HDL‐C (OR: 1.76, 95% CI: 1.34–2.18, *p* = 0.023) and BMI (OR: 1.73, 95% CI: 1.28–2.18, *p* = 0.036) weakened such associations, separately (Table 2).

**TABLE 2:** The relationship between the level of serum uric acid and carotid plaque

Items	OR	95% CI	*p* value
Univariate	2.01	1.83–2.19	0.003
Model 1	1.95	1.81–2.09	0.013
Model 2	1.83	1.56–2.11	0.025
Model 3	1.76	1.34–2.18	0.023
Model 4	1.73	1.28–2.18	0.036

Note

Model 1, adjusting the age; Model 2, adjusting additional adjustments for smoking, drinking, hypertension, diabetes mellitus, and the drug history of statin, antihypertensive medication, antidiabetic medication, and HRT; Model 3, additional adjustments for TC, TG, LDL‐C, and HDL‐C; Model 4, additional adjustments for BMI.

Abbreviations: CI, confidence interval; OR, odds ratio.

### 3.3 The level of SUA and the progression of CAS

The relationship of the change of plaque score and the change of carotid intima‐media thickness with the level of SUA is shown in Tables 3 and 4, respectively. In the univariate linear regression analysis, a 1 mg/dL increase in SUA levels was expected to 0.946% increase in plaque score. After adjusting the age, such association remained significant but mildly decreased (β: 0.838, 95% CI: 0.405–1.271, *p* = 0.021). And additional adjustments for smoking, drinking, hypertension, diabetes mellitus, and the drug history of statin, antihypertensive medication, antidiabetic medication, and HRT wakened such associations as well (β: 0.769, 95% CI: 0.373–1.165, *p* = 0.037). Further adjustments for TC, TG, LDL‐C, HDL‐C, and BMI also attenuated the associations.

**TABLE 3:** The relationship between the level of serum uric acid and the change of plaque score of carotid atherosclerosis

Items	β	95% CI	*p* value
Univariate	0.946	0.512–1.380	0.009
Model 1	0.838	0.405–1.271	0.021
Model 2	0.769	0.373–1.165	0.037
Model 3	0.742	0.329–1.155	0.039
Model 4	0.659	0.298–1.011	0.042

Note

Model 1, adjusting the age; Model 2, adjusting additional adjustments for smoking, drinking, hypertension, diabetes mellitus, and the drug history of statin, antihypertensive medication, antidiabetic medication, and HRT; Model 3, additional adjustments for TC, TG, LDL‐C, and HDL‐C; Model 4, additional adjustments for BMI.

Abbreviation: CI, confidence interval.

**TABLE 4:** The relationship between the level of serum uric acid and the change of carotid intima‐media thickness

Items	β	95% CI	*p* value
Univariate	0.026	0.018–0.034	0.023
Model 1	0.022	0.016–0.028	0.026
Model 2	0.017	0.009–0.025	0.037
Model 3	0.010	0.004–0.016	0.036
Model 4	0.008	0.002–0.014	0.049

Note

Model 1, adjusting the age; Model 2, adjusting additional adjustments for smoking, drinking, hypertension, diabetes mellitus, and the drug history of statin, antihypertensive medication, antidiabetic medication, and HRT; Model 3, additional adjustments for TC, TG, LDL‐C, and HDL‐C; Model 4, additional adjustments for BMI.

Abbreviation: CI, confidence interval.

In terms of the change of carotid intima‐media thickness, a 1 mg/dL increase in SUA levels was expected to 0.026 cm increase in carotid intima‐media thickness (95% CI: 0.018–0.034, *p* = 0.023). And when we adjusted the confounding factors mentioned above, their associations weakened but remained statistically significant differences.

### 3.4 The effect of HRT on elderly postmenopausal women with plaques

According to the medication histories provided by these elderly postmenopausal patients with plaques, there were 214 patients who received long‐term HRT (≥5 years), and they were delimited to the HRT group. Table 5 shows the comparisons of age, BMI, SUA, the change of plaque score and carotid intima‐media thickness, the serum lipid data between the HRT groups, and the non‐HRT group. It revealed that the female patients with plaques did not have a significant difference in age between the two groups, but the BMI, SUA, the change of plaque score, carotid intima‐media thickness, and the serum lipid data exhibited statistically significant differences. The elderly postmenopausal patients with plaques who received long‐term HRT had lower BMI, the levels of SUA, TC, TG, and LDL‐C and higher levels of HDL‐C than the patients who never received HRT. Most importantly, the change of plaque score and carotid intima‐media thickness was significantly less in the HRT group than in the non‐HRT group (*p* < 0.05).

**TABLE 5:** The effects of hormone replacement therapy on the level of serum uric acid, the serum lipid concentrations, and the carotid intima‐media thickness

Variables	No HRT (*n* = 254)	HRT (*n* = 250)	*p* value
Age	73.12 ± 3.65	72.67 ± 3.72	0.328
BMI, kg/m^2^	24.65 ± 3.29	23.07 ± 3.31	0.034
SUA, mg/dL	7.46 ± 2.46	6.95 ± 2.43	0.041
Percentage of change of plaque score, %	10.32 ± 4.17	7.65 ± 5.31	0.028
Change of CIMT, cm	0.043 ± 0.009	0.011 ± 0.006	0.047
TC, mg/dL	232.65 ± 15.37	225.42 ± 12.45	0.029
TG, mg/dL	95.78 ± 23.32	92.74 ± 20.45	0.031
LDL‐C, mg/dL	105.46 ± 19.76	98.42 ± 17.92	0.002
HDL‐C, mg/dL	72.93 ± 21.36	83.45 ± 19.65	0.003
Use of statin	46 (23.2%)	52 (24.3%)	0.799

Abbreviations: BMI, body mass index; CIMT, carotid intima‐media thickness; HDL‐C, high‐density lipoprotein cholesterol; HRT, hormone replacement therapy; LDL‐C, low‐density lipoprotein cholesterol; SUA, serum uric acid; TC, total cholesterol; TG, triglyceride.

## 4 DISCUSSION

In this study, we found that the level of SUA had significantly positive associations with the presence and progression of CAS in postmenopausal women aged over 70 years. And the long‐term HRT may decrease SUA and blood lipid levels and reduce the change of plaque score and carotid intima‐media thickness. Therefore, this study suggested that SUA might bear a good predictive and prognostic value for CAS.

Uric acid is the metabolic product of purine breakdown, which keeps in balance with its disposal under the steady‐state condition.^16^ As a ubiquitous metabolite appeared in serum, its elevation was obviously associated with the risk of many cardiovascular diseases.^17^ Our study investigates the relationship between SUA and atherosclerosis in elderly postmenopausal women and found the SUA level had a significantly positive association with the presence and progression of CAS, which was similar to previous studies. For instance, a study conducted on Japanese individuals revealed that SUA might be an independent risk factor for the incidence of CAS for males without metabolic syndrome.^18^ Another study conducted in Italy found that increased SUA levels are associated with CAS in obese children and adolescents.^19^ Moreover, a study conducted on healthy individuals showed that increased SUA levels, even in the physiological range, could increase the risk of aortic stiffness as an independent factor.^20^ These researches demonstrated that the measurement of SUA levels of patients might benefit the risk prediction of CAS.

Although this study revealed the association between SUA and atherosclerosis in postmenopausal women aged over 70 years, whether the SUA is a cause or consequence of CAS remains controversial. Elevated SUA levels were related to many established cardiovascular risk factors, such as metabolic syndrome, hypertension, and renal disease.^21^ However, the participants enrolled in our study were elderly people who may be suffered from these diseases, which might result in the multicollinearity of our models. Although our study adjusted many potential confounding factors (Tables 1, 2, 3, 4), such as age, smoking, drinking, hypertension, diabetes mellitus, and drug histories, the coexistence of multiple diseases associated with the increased SUA should be considered in further studies.

This study also found that patients treated by long‐term HRT may have a lower level of SUA and blood lipid and less change of plaque score and carotid intima‐media thickness than patients without HRT (Table 5). It was consistent with previous studies that showed that estrogen and sex hormone‐binding globulin (SHBG) were related to the reduced progression of SA in healthy postmenopausal women.^22^, ^23^ And studies suggested that the effect of HRT on the presence and progression of SA may result from its regulation of lipoprotein metabolism.^24^ Moreover, previous researches conducted on postmenopausal women with hyperuricemia and found that long‐term HRT could significantly decrease the mean concentration of SUA.^25^, ^26^ Therefore, we assumed that HRT could prevent the presence and progression of SA by reducing SUA concentrations. Unlike the CAS, HRT seems not to exert benefits for older postmenopausal women with established coronary‐artery atherosclerosis. A double‐blind, placebo‐controlled trial has shown that no significant effect of 17β‐estradiol either alone or with sequentially taken medroxyprogesterone acetate was found on the progression of coronary atherosclerosis for postmenopausal women.^27^ Another randomized controlled trial also suggested that HRT did not provide cardiovascular benefit in postmenopausal women with coronary disease, but a harmful effect might be for them instead.^28^ Furthermore, other studies also indicated that starting HRT may slightly increase the risk of cardiovascular disease in older postmenopausal women, and the overall lifetime occurrence rate has no obvious change.^29^ Thus, due to the multiple side effects of HRT, including an increased risk of cancer, we took the view that HRT should be applied to elderly postmenopausal women only for symptomatic treatment rather than the prevention of the presence and progression of atherosclerosis.^30^

Although this study enriched the evidence of the association between serum uric acid and atherosclerosis in elderly postmenopausal women, several limitations should also be mentioned. Firstly, our study was a single‐center study, so the samples were still relatively insufficient. Further multicenter prospective studies should be conducted to verify our conclusion. Secondly, although we have adjusted for some confounding factors, further studies should consider some diseases which may cause multicollinearity. Thirdly, the mechanism of the HRT on CAS should also be investigated in further studies. And the long‐term outcomes and costs should also be concerned.

## 5 CONCLUSION

In conclusion, this study suggested that increased SUA level was significantly associated with the presence and progression of CAS in postmenopausal women aged over 70 years. And the HRT may exert benefits for them by preventing the presence and progression of CAS. However, the safety and long‐term outcome of HRT on CAS should be evaluated in further studies.

## CONFLICT OF INTEREST

None.

## Data Availability

The datasets used and/or analyzed during the current study available from the corresponding author on reasonable request.
